# Past and future uses of text mining in ecology and evolution

**DOI:** 10.1098/rspb.2021.2721

**Published:** 2022-05-25

**Authors:** Maxwell J. Farrell, Liam Brierley, Anna Willoughby, Andrew Yates, Nicole Mideo

**Affiliations:** ^1^ Department of Ecology and Evolutionary Biology, University of Toronto, Toronto, Canada; ^2^ Department of Health Data Science, University of Liverpool, Liverpool, UK; ^3^ Odum School of Ecology, University of Georgia, Athens, GA, USA; ^4^ Center for the Ecology of Infectious Diseases, University of Georgia, Athens, GA, USA; ^5^ University of Amsterdam, Amsterdam, The Netherlands

**Keywords:** natural language processing, literature synthesis, computational linguistics, information extraction, database construction

## Abstract

Ecology and evolutionary biology, like other scientific fields, are experiencing an exponential growth of academic manuscripts. As domain knowledge accumulates, scientists will need new computational approaches for identifying relevant literature to read and include in formal literature reviews and meta-analyses. Importantly, these approaches can also facilitate automated, large-scale data synthesis tasks and build structured databases from the information in the texts of primary journal articles, books, grey literature, and websites. The increasing availability of digital text, computational resources, and machine-learning based language models have led to a revolution in text analysis and natural language processing (NLP) in recent years. NLP has been widely adopted across the biomedical sciences but is rarely used in ecology and evolutionary biology. Applying computational tools from text mining and NLP will increase the efficiency of data synthesis, improve the reproducibility of literature reviews, formalize analyses of research biases and knowledge gaps, and promote data-driven discovery of patterns across ecology and evolutionary biology. Here we present recent use cases from ecology and evolution, and discuss future applications, limitations and ethical issues.

## 1. Why use text mining?

The volume of scientific literature is growing exponentially [1], with over three million peer-reviewed academic articles published each year [2]. In a sample of 33 ecology journals alone, over 80 000 articles have been published since 1980 [3]. Reading this amount of material is an insurmountable task, making manual literature syntheses and compilation of literature-based datasets increasingly difficult. As bodies of literature continue to grow, highly cited papers are more likely to be cited compared to recent work, which can result in slowing of scientific progress as transformative ideas are less likely to permeate and make substantive impact [4]. Adopting computational approaches for analysis of scientific texts allows researchers to rapidly and systematically identify relevant publications and synthesize larger amounts of literature compared to manual approaches. Beyond literature syntheses, computational tools can be used to efficiently extract information from texts and update existing literature-based databases, ultimately increasing the value of published research.

When humans read, we interpret information in text through the meaning of words and grammatical contexts. To a computer, human language is complex and difficult to convert to structured formats, such as tabular or relational databases commonly used in scientific research. Therefore, raw text is commonly referred to as ‘unstructured’. To convert unstructured data in scientific texts to a format ready for statistical analysis, we can apply a diverse set of computational approaches. These tools broadly fall under the umbrella of ‘text mining’, but often come from natural language processing (NLP), a field that focuses on computational interpretation of human language, blending theory and approaches from linguistics, computer science, statistics and artificial intelligence. NLP comprises an extremely broad set of computational methods that allow us to gather, sort, translate and understand written documents.

**Figure 1.**
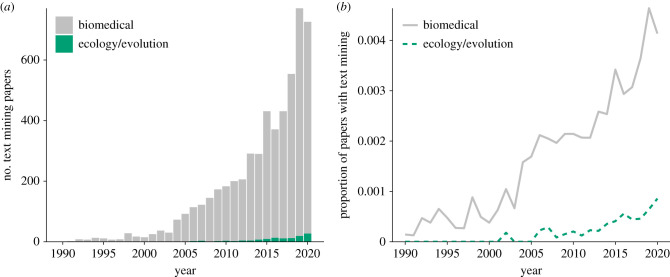
Publication trends indicating an earlier adoption, and greater (*a*) absolute number and (*b*) proportion of papers involving text mining in biomedical publications compared to ecology and evolutionary biology. Data were from two Web of Science (WOS) searches: one with ‘*medic*’ and the other with ‘ecology’ OR ‘evolutionary biology’ OR ‘biodiversity’ in the Topic field, plus ‘text mining’ OR ‘Natural Language Processing’ OR ‘NLP’ in All Fields for each search. A total of 5262 biomedical papers and 120 ecology/evolutionary biology papers mentioning text mining or NLP were identified out of a total 2 355 632 biomedical and 354 798 ecology/evolution papers. Searches were conducted on 10 September 2021 via the University of Toronto subscription. Note that variation in WOS search results varies owing to institutional subscriptions [10]. Search results were subset to the years 1990–2020 inclusive. Data and R code to reproduce the figure, and .bib files with citation information for the returned articles can be accessed at https://github.com/maxfarrell/textmining_trends. (Online version in colour.)

Tools for mining scientific texts have seen wide-scale adoption in other fields, such as biomedical sciences, where models have been developed to recommend relevant literature and extract data for further analysis. Exciting examples include the construction of large-scale databases of protein–protein interactions [5], drug–drug interactions [6], gene-disease relations [7], chemical-disease relations [8], and interfaces to extract information using structured searches [9]. Applications of NLP in ecology and evolution are relatively rare compared to biomedical sciences (figure 1). The disparity in onset and magnitude of adoption suggests that ecology and evolution researchers could look to biomedical studies for inspiration on applying classical and cutting edge NLP approaches in their projects.

NLP itself is a rapidly growing field with many approaches applicable to ecology and evolution. In recent years, ecologists and evolutionary biologists have begun to develop similar domain-specific approaches, but their applications have largely been restricted to the analysis of publishing trends and related metrics. Given the growing and diverse types of literature, the importance of research syntheses, and increasing computational literacy in the field, ecology and evolutionary biology are prime candidates for the application of more advanced text mining and NLP approaches.

Using NLP to create literature-based databases holds particular value for comparative studies and biodiversity syntheses as these projects can be greatly accelerated by improving the reproducibility and efficiency of data integration [11]. Further, the aggregation of key biodiversity data enables analyses that would not otherwise be possible [12]. While peer-reviewed literature in journals represents the most common source of scientific texts, application of NLP to other texts, such as preprints [13], can highlight emergent and rapidly changing science, such as the COVID-19 pandemic [14,15]. Considerable ecological knowledge is also stored in older books and texts associated with archival samples and natural history collections [16], but recent advances in document scanning, digitization, and optical character recognition (e.g. from printed or handwritten texts) mean NLP approaches are now feasible and promising [17,18]. This technological advancement parallels the invention of new sensors and machine learning tools for image analysis in wildlife conservation [19,20]. Similarly, there exist vast amounts of text published alongside online genetic sequence databases such as GenBank or the Gene Expression Omnibus [21]. With increasing digitization efforts and availability of associated texts, adoption of text mining in ecology and evolution could greatly expand metadata and maximize the use of these ever-growing resources.

Beyond supporting the efficient creation and expansion of literature-derived databases, using scripted and archived computational processes for text analysis can dramatically improve transparency, and help the reproducibility in all phases of research, from identifying relevant papers, analysing research trends, constructing and expanding datasets and automated translation of text into data ready for statistical analysis. Here we outline current and future applications of text mining in ecology and evolutionary biology and discuss current barriers to implementation.

## 2. Recent applications in ecology and evolution

### (a) Detecting trends and topics

The most common uses of text analysis in ecology and evolution have been under the umbrella of bibliometrics: quantitative research that studies trends in subject matter, authorship, and impact of publications. For example, Anderson *et al*. [22] analysed over 130 000 articles to explore the increasing diversity of ecological hypotheses and theories published over the past 80 years. Similar studies of publishing trends have explored ecological topics in high impact journals [23], showed the emergence of conservation biology as a separate discipline from ecology [24], analysed the growth of interdisciplinarity in biodiversity science [25], tracked shifting popularity of topics within industrial ecology [26] and fish ecology [27], identified research themes in disease ecology [28], and pinpointed critical research gaps in conservation science [29] and pollination ecology [30]. Outside of academic articles, text mining can reveal important trends for environmental management and biodiversity conservation [31]. In conservation science, analysis of online texts and social media posts led to the development of *conservation culturomics*, a field that evaluates public interest in nature [32], tracks opinions on conservation topics [33] and quantifies people's experiences in nature [34] based on an increasingly diverse set of data sources [35]. Beyond tracking trends, text analysis can be used to gather evidence supporting the success of conservation actions and develop more culturally relevant policies.

### (b) Evidence synthesis and literature reviews

The growth of scientific literature is making evidence synthesis an increasingly difficult task, leading to an ever-widening ‘synthesis gap’ [36]. For both narrative and systematic reviews, text mining is projected to become a necessary tool to circumvent literature overload [37]. Text analysis can be implemented at multiple phases of a review, from identifying search terms using keyword co-occurrence networks [38], to applying predictive approaches to screen studies for inclusion [36]. Abstract screening using text mining and machine learning can be a precise and efficient alternative to the common practice of screening abstracts with two reviewers [39], which may help limit individual biases by providing a consensus annotation, but is time consuming and can be error-prone. The future of systematic reviewing will necessitate the interaction of humans and machine learning algorithms to tackle the rapid growth in publications [40]. Overall, implementing computational processes can dramatically expand literature assessments to include more diverse texts, increase the efficiency of reviews and literature syntheses, and allow rapid reproducibility and updating as new literature is published [37]. These tools need to be properly calibrated and validated to ensure accuracy compared to manual search and screening [36,41,42].

### (c) Expanding literature-based datasets

Large-scale studies in ecology are often based on data compiled from previously published research and typically involve significant manual investment for literature searching, acquisition, screening, data extraction, and harmonization of entities such as species names, place names, measurement units, experimental designs and terminology with inconsistent definitions [12]. As such, these studies require substantial effort to update as new papers are published. In NLP, the sub-field of information retrieval develops search algorithms and models that suggest articles of potential interest. In a recent ecological application, Cornford *et al*. [43] train machine learning models to classify literature as relevant to the PREDICTS database [44], a literature-based database of biodiversity responses to human impacts. Their best models could distinguish relevant from non-relevant articles with over 90% accuracy based only on title and abstract text, significantly improving the speed and ease with which new articles can be screened for database inclusion. A similar machine learning approach was used by Roll *et al*. [45] to identify articles using the term ‘reintroduction’ in a conservation context (release of organisms into their historical native habitat), rather than a non-ecological context. Outside of search engines, a number of machine learning models for text classification have been developed in recent years [46], but are rarely used in ecology and evolutionary biology [47]. The ability to continually flag and integrate relevant publications will help transition from static ecological datasets to living ones, and help promote more efficient, timely, and impactful science.

### (d) Extraction and integration of primary biodiversity data

Integrating data from across the life sciences is currently a major challenge, but will foster the interdisciplinary research needed to address pressing global issues [48]. With NLP approaches, unstructured texts can be more efficiently transformed into structured data commonly analysed in ecological and evolutionary studies. With dictionaries containing terms of interest (e.g. species names, traits, keywords describing an ecological interaction), the frequency of term co-occurrences can be used to discover associations [49]. For example, by quantifying the co-occurrence frequencies of ant species names and terms describing ant-plant mutualisms, Kaur *et al*. [50] were able to identify ant species associated with mutualistic behaviours, and used the compiled dataset to study the evolution of plant mutualisms. Similar approaches have been used to infer inter-species associations via descriptions from the Encyclopedia of Life [51], and NCBI and PubMed [52,53]. Ecologists have also used text from Twitter [54] and news sources to gather species-linked data that can infer population trends, geographical ranges or even monetary values, that support innovative systems to monitor and respond to conservation concerns [55].

These studies used dictionaries to identify relevant terms, but to go beyond lists of words, terms can be linked to other datasets using ontologies. In linguistics, an ontology refers to a set of terms and their relationships, forming a network of concepts in a domain [56]. Ontologies capture expert knowledge and allow users to translate concepts across databases, disambiguate terms with different disciplinary meanings, or collapse terms into larger concepts (much like a taxonomy allows collapsing species into genera, families, orders, etc…). Ontologies have proven useful in biomedicine [57,58] and for harmonizing data across diverse texts to study problems within environmental science, bacterial evolution, and comparative anatomy [59–64].

Ecology and evolution are rife with ambiguously defined terminology (e.g. the definition of ‘virulence’ depends on if the pathogen infects a plant or animal host, and often differs between theory and empirical papers [65]), which slows research progress and limits the ability to synthesize across studies [66,67]. Creating platforms with consistent naming conventions and connected concepts will facilitate data harmonization, sharing and annotation and aid collaborative research projects already common in biodiversity science [68]. There exist a number of related ontologies describing ecological observations [69], biological collections [70], phenotypes [71,72], and biodiversity science [73]. Recent efforts have aimed to generate consensus definitions for ecological traits with ontologies [74]. These act as resources for describing, accessing and manipulating phenotypic data by making phenotypic data more manipulable by computers [75], efficiently extracting phenotypic data from taxonomic descriptions [76], structuring species information [77], and harmonizing traits with taxa [78]. Developing diverse vocabularies, definitions and relationships among concepts is crucial for dealing with the heterogeneous nature of information in ecology and evolution, and these initiatives will lay the groundwork for more automated text analyses in the future.

## 3. Future uses of text mining and natural language processing in ecology and evolution

**Figure 2.**
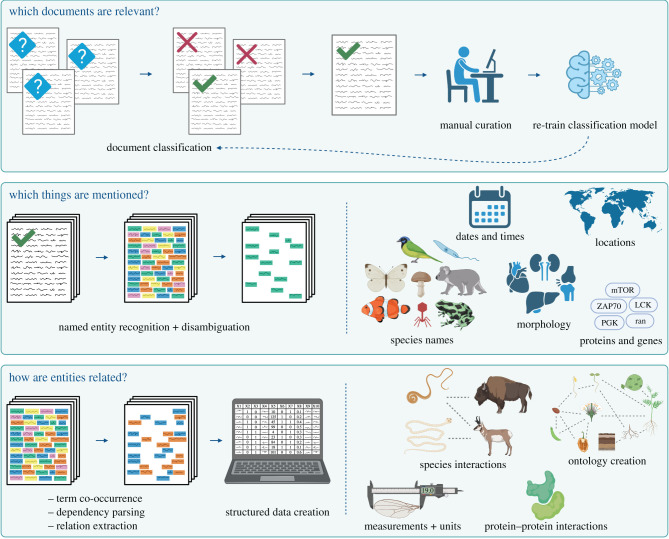
Potential applications of natural language models in ecology and evolution. The simplest application is training and applying a document classifier to predict relevant documents (top row). Given a training set of relevant and non-relevant documents (may come from existing databases, a manually curated training set, or documents tagged by a set of rules), the relevance of new documents may be predicted and prioritized for manual screening and curation, or downstream information extraction. Manual screening may be used to validate predictive models or re-train and fine-tune the original classifier. Once a set of relevant documents is identified, the subjects of the documents can be explored through named entity recognition (NER; middle row). Named entities can be identified by comparing text strings to a dictionary. If a complete set of entities is not known or available, a machine learning-based NER tool can be used to predict entities and identify never-before-seen terms. Given a training set, NER can be used to identify terms in a text (for example, species, genes, proteins, locations, morphological structures) and tag their locations in a text. Once components of a document are tagged (parts of speech, named entities, numbers), relationships among them can be identified to create structured datasets for analysis (bottom row). Relationships may be inferred through term co-occurrence frequencies, sentence structures (dependency parsing), or through machine learning-based models that predict the nature of the relationship. Relational data can take a variety of forms including species interactions, biological measurements and their associated units, or networks of different relationship types (ontologies). Figure created with BioRender.com. (Online version in colour.)

Given the current limited use of NLP approaches in ecology and evolution, we suggest that their adoption will have the greatest impact on the construction of large scale comparative databases. We highlight three tasks that are likely to be extremely useful: document classification, tagging domain-specific entities in text, and building structured databases through relation extraction (figure 2). Each of these tools can be generalized to future research projects, or linked together to build a workflow from raw texts to a structured database ready for analysis. In general, model performance will differ based on the specific task, goals of the larger project, and to what degree metrics such as precision or recall should be optimized. For example, a computational approach may not return all articles identified in a manual search, but may still be desirable if it identifies a larger number of relevant texts to include, or offers the ability to more rapidly analyse a larger set of documents. Below we assume that some source texts (corpus) have already been identified, either through targeted literature searches, or choice of an existing body of literature. We do not discuss article search strategies, as detailed guides exist [79], but note that this is an important consideration when gathering a corpus and designing a text mining project.

### (a) Document classification

The success of document classification by Cornford *et al*. [43] demonstrates the potential for document-level predictive models to aid the updating of large-scale comparative databases. As a general template, if databases derived from published articles can be linked with abstracts or full texts, classifiers can be trained to predict whether subsequently published articles are likely to contain relevant data. Training a classifier requires examples of both positive and negative cases (e.g. relevant and irrelevant articles). Databases that report discarded literature are great resources of positive and negative examples. However, because existing databases rarely document these, ‘irrelevant’ papers may be identified by sampling papers in the discipline, such as the use of general ecology papers by Cornford *et al*. [43]. These irrelevant articles are similar to the use of ‘background’ or ‘pseudo-absence’ data in species distribution models [80] in that they may contain undocumented positives (i.e. relevant articles), but the assumption is that the majority will be irrelevant and provide a useful contrast to those in existing databases.

The choice of negative examples for training should reflect future search strategies, whether it be searching through all ecology papers, or a more specific set. If the source database clearly outlines their strategy for literature inclusion (i.e. search terms, targeted journals, publication dates), it may be possible to compile more targeted sets of negative examples for training. Future development of document classifiers should explore the influence of these different approaches for generating negative training data on accuracy, and validate these predictive models on articles which have been expert-validated rather than assumed to be irrelevant (figure 2). In addition to periodic updating, using predictive models to expand existing datasets will lay the foundation for systems that can alert researchers of relevant papers as they are published, and automatically extract data from these papers.

### (b) Identifying entities specific to ecology and evolution

Once relevant texts are identified, the next task is extraction of relevant terms. If researchers know exactly what they are looking for, and terms of interest are completely known and can be listed, simple methods such as text matching can be used to identify them. However, given the diversity of specialised terms in ecology, this is unlikely to be the case. When relevant terms are not known, or texts are expected to include never-before-seen terms, named entity recognition (NER) will be extremely useful. NER involves identifying real-world objects (named entities) based on the context of their surrounding text, such as people, locations, organizations, etc. In biomedical text analysis, specialized NER tools are built to identify mentions of diseases, genes, proteins, cell types, and chemicals [81]. NER tools designed for ecology and evolutionary biology are currently rare, but would greatly improve literature exploration and information extraction. Contemporary NER tools are often created by adapting deep learning based language models [82]. Therefore, given suitable training data, NER models can be trained to recognize and disambiguate ecology-specific entities (figure 2). For example, the recently developed TaxoNERD [78] is a deep-learning based model that recognizes scientific and common species names, and can normalize names to match NCBI or GBIF. One current challenge to developing deep learning-based ecological language models from scratch is the lack of domain-specific ‘gold standard’ training data. However, the authors of TaxoNERD overcome this by starting with a pre-trained biomedical language model and updating it for an ecological task. This successful example of transfer learning demonstrates the potential of large deep-learning based models to generalize to novel tasks and reduce the amount of labelled training data needed to build a tool explicitly for ecology and evolutionary biology. Moving forward, the development of NLP tools for ecology and evolution could be greatly supported by hubs of open access training data, such as those created for image analysis in biology and conservation [83].

Once named entities are recognized, a text analysis pipeline can take many different paths. To better understand context, researchers may cross-reference terms with ontologies to connect concepts or collapse terms into higher groups. For example, scispaCy v. 2.5.0 supports entity linking to biomedical ontologies including the Unified Medical Language System (UMLS) [84] and the Medical Subject Headings (MeSH terms) [85], which in turn allow them to be connected to a diverse array of databases. These may be used to group organs into larger anatomical systems, or categorize proteins into enzymes, hormones, or antibodies. While approaches have been developed to identify taxonomic, morphological and habitat entities [63,86], merge existing ontologies [87,88] and create standards for publishing of biodiversity information [89], these initiatives remain disconnected, and have not yet been integrated with contemporary NLP software.

### (c) Relation extraction and creation of structured datasets

Once entities are recognized, and disambiguated or linked to an ontology, multiple approaches can be used to identify relationships among these entities (for examples, figure 2 and table 1). One approach is analysis of term co-occurrences, as used by Kaur *et al*. [50] to identify ant-plant mutualisms. Alternatively, the structure of the text itself can be used to identify the relationships, through a task referred to as relation extraction. Relation extraction can be done by incorporating linguistic information, such as semantic relationships between entities, or through training of a deep-learning based language model if one is available. For example, identifying protein-protein interactions in text has progressed from using a dictionary of protein names and co-occurrences, to adding information about parts of speech (e.g. verbs, nouns, adjectives), to supervised and deep learning approaches that incorporate vector representations of articles as predictors [90]. Relation extraction can also be used to identify relationships between different classes of entities, such as disease-gene interactions [7]. Relation extraction is often a complex task, which can be daunting for researchers new to text mining. However, given the diversity and value of relational data in ecology and evolution, we suggest that relation extraction will be an increasingly important means of generating structured, analysis-ready data in the future. This offers exciting new frontiers for ecologists and evolutionary biologists to collaborate with computational linguists and computer scientists.

**Table 1.** Table of common relationship types in ecology and evolution, and example texts. Italics are species names, underlining are the entities, bold are the relations.

example of relation	example text
measurements and units	‘The average length of human gestation **is** 280 days'
model-specific parameters	‘R_0_ was **estimated** to be 1.13’
species interactions	‘*Anoplocephala manubriata* **parasitizes** Asian elephants’
protein–protein interactions	‘Pleiotropic drug resistance 1p (Pdr1p) **regulates** Pdr5p’
habitat associations	‘*Ribes mandschuricum* is **found in** shady areas’
species occurrences	‘Cercopia moths were **collected from** sites throughout Massachusetts ‘
Linnaean taxonomy/common names/synonyms	‘*Boops boops*, **commonly called** the bogue, is a species of seabream native to the eastern Atlantic’
anthropogenic impacts	‘*Inversodicraea botswana* is **threatened by** sewage discharge’

## 4. Current barriers to adoption and pathways forward

Despite the promise of text mining to revolutionize literature synthesis and database creation, several technical and social barriers currently limit widespread adoption in ecology and evolution. These include a lack of knowledge of existing tools, best practices, and shared vocabularies needed for collaboration with computational linguists [36]. Further, there are inequalities in access to software, data, and academic literature [10,47,91]. To use text mining and NLP in ecology and evolution to their full potential, we need to promote awareness of these methods, improve access to scientific literature and article-level metadata, facilitate cross-disciplinary collaborations, create domain-specific software, and develop an ecosystem of scientific language tools that work across all the world's languages, not just English. Recent successful applications of NLP approaches in ecology and conservation biology have involved close collaborations between biologists and computer scientists [55,92] highlighting the importance of cross-disciplinary research. However, as general tools and frameworks exist, their adoption in ecology and evolution is now limited by access to texts, development of applications specific to ecology, and the dissemination and uptake of these tools.

For primary literature, abstracts are among the most readily accessible documents and can be sufficient for document classification and database creation [43,50,93]. However, abstracts may not be available for more historical papers [22], and analyses of manuscript full texts are likely to outperform the use of abstracts only, as shown for relation extraction [93]. Unlike abstracts, access to full academic texts is limited by institutional subscriptions [10], with only half of publishers releasing manuscripts in a machine readable format [94]. Access to paywalled articles and copyright issues will limit the reproducibility of studies using text mining, and re-publishing or hosting source texts as supplementary materials may be illegal. Projects such as the PMC Open Access Subset offers bulk download of 100 000s of articles in machine-readable format [95], and The General Index [96], an open access database of text sequences and keywords extracted from 107 million journal articles, offers researchers the ability to perform specialized searches and analyse thematic trends in scientific literature without barriers imposed by paywalls or institutional access. While such databases can greatly improve interaction with published literature, their success relies on unrestricted bulk access to primary texts. Interfaces such as application programming interfaces can facilitate scripted retrieval of texts, but usually involve arbitrary rate-limitation which makes large-scale analyses difficult and hampers literature-based research [97]. Thus, scientific advances in synthesizing studies in ecology and evolution are limited by business decisions and publisher-imposed restrictions that create artificial scarcity [98]. In turn, when analysing large volumes of papers, researchers should take care to cite primary sources appropriately. However, the mainstreaming of text mining has resulted in a need for new bibliometric and citation infrastructure to facilitate transparent and permanent linking of large citation lists, and allow proper acknowledgement of individual studies that underlie large-scale literature surveys. Overall, the scale and reproducibility of text mining studies will be hindered until scientific articles are considered a public good and made open and freely accessible.

Parallel to variation in access to scientific publications, the dominance of English in science has led to data from non-English publications being omitted from ecological syntheses [99]. There also exist systematic inequalities in the representation and performance of NLP technologies across languages [91,100]: largely because of the historical dominance of English as the *lingua franca* of scientific publishing, current scientific language models are designed only for English texts [101,102]. As training data and models for previously under-supported languages continue to grow [103], the future looks promising for expansion of NLP approaches to non-English scientific texts. This could promote broader inclusion in science by facilitating translation of publications across languages, easing barriers for researchers to publish in their chosen languages, and allowing broader inclusion of non-English scientific texts in synthetic research.

## 5. Conclusion

The application of text mining and natural language models to domain-specific text in ecology and evolutionary biology shows great promise for summarizing historical research and current gaps in knowledge, efficiently identifying pertinent literature, constructing structured databases from unstructured texts and developing real-time biodiversity surveillance for issues such as emerging diseases and conservation threats. We urge early-career scientists and established researchers alike to explore and apply these tools in their own research, foster interdisciplinary collaborations, build open access corpora, contribute their expertise to developing open-source software and expert-created training data, and develop tools that are designed specifically for processing texts in ecology and evolution.

## Data Availability

We create a very simple graph of number of publications through time. We detail the exact search strategies in the figure caption, so as to make this search reproduced by readers. We also provide a link to a github repository with the underlying data and R script used to make this figure, and reference this in the caption of figure 1 (https://github.com/maxfarrell/textmining_trends).
